# Magnitude of, trends in, and associated factors of road traffic collision in central Ethiopia

**DOI:** 10.1186/1471-2458-14-1072

**Published:** 2014-10-15

**Authors:** Fekede Asefa, Demeke Assefa, Gezahegn Tesfaye

**Affiliations:** Department of Public Health, College of Health and Medical Sciences, Haramaya University, P.O. Box 235, Harar, Ethiopia; School of Public Health, College of Health Sciences, Addis Ababa University, P.O. Box 19304, Addis Ababa, Ethiopia

**Keywords:** Magnitude, Trends, Road traffic collision, Road traffic collisions fatality

## Abstract

**Background:**

Road traffic collision (RTC) is one of many public health problems. Globally, about 1.2 million people die due to RTCs every year. Of these, 85% reside in low- and middle-income countries. Despite low road network density and vehicle ownership, Ethiopia has a relatively high collision record. Collisions in the Addis Ababa and Oromia Regions account for 58% of all fatal collisions in Ethiopia. The aim of this study was to assess the magnitude of, trends in and factors associated with RTCs in central Ethiopia.

**Methods:**

A retrospective study was conducted using relevant police reports obtained from eight police stations found between Akaki and Adama towns located in central Ethiopia. The study included reports from July 2007 to June 2012. Both quantitative and qualitative techniques were employed, and bivariate and multivariate analyses were performed to identify the factors associated with the RTCs.

**Results:**

From July 2007 to June 2012, 2,335 collisions were registered, though the outcomes of 24 of these crashes were not recorded. Among these collisions, 389 (16.7%) resulted in death, 316 (13.5%) brought about severe injuries, 290 (12.4%) caused slight injuries, and 1,316 (56.4%) caused property damage. These collisions affected about 1,745 individuals. While 515 (29.5%) people died, 549 (31.5%) were severely injured, and the remaining 681 (39%) were slightly injured. Driving at midnight [AOR 1.67, 95% CI; 1.2-2.4], driving above the speed limit [AOR 5.3, 95% CI; 2.9-9.6], failing to give priority for other vehicles and pedestrians [AOR 5.03, 95% CI; 2.3-9.3], and vehicular technical problems [AOR 19, 95% CI; 6.4-56] were determinants of RTC fatality.

**Conclusions:**

RTCs steadily increased in the study area over this period of time. This calls for urgent interventions. Ensuring that drivers obey traffic rules and enforcing the speed limit appear to be the most critical parts of necessary interventions.

## Background

Of all the transportation systems that people use, road transport is the most widely used, complex and dangerous because it is highly associated with the rise in road traffic collisions (RTC) [1]. RTC can be defined as a “*collision that occurs on a way or street open to public traffic, results in one or more persons being killed or injured, and at least one moving vehicle is involved. Thus, RTC is a collision between vehicles, between vehicles and pedestrians, between vehicles and animals, or between vehicles and geographical or architectural obstacles*” [2].

According to the World Health Organization (WHO) (2004), globally, more than 1.23 million people die due to RTC every year, while the number of injured is as high as 50 million. If trends in RTCs continue as they are now, it is estimated that road traffic deaths and injuries could rise 65% by 2020. Further, most of the deaths and injuries (80%) will occur in low- and middle-income countries [1, 3]. In Africa, 28.3 per 100,000 die in collisions. In Ethiopia, a country with a small vehicle-population ratio, 95 deaths per 10, 000 vehicles were registered between 2007 and 2008 [4]. While this is the highest RTC rate among African countries, little research has been carried out on the causes of collisions in the country. Moreover, Ethiopia is currently labeled as one of the most unsafe places to drive. Road safety has become a concern of government because of the need to address the worsening situation in RTC deaths, injuries and property loss. Thus, well-conducted, scientifically rigorous research on the burden of RTCs, risk factors and effectiveness of interventions are crucial elements that need to be prioritized in order to prevent and control RTCs [5]. Therefore, the aim of this study was to assess the magnitude of, trends in and associated factors of RTCs in central Ethiopia.

## Methods

### Study setting and design

The study was conducted between Akaki and Adama towns, located in central Ethiopia, which has the highest traffic movement in the country, next to the capital city of Addis Ababa. Between Akaki and Adama towns, there are a number of towns, including Gelan, Dukem, Bishoftu, and Mojo. The road between Akaki and Adama is about 100 Km and connects the capital city to the Southern and Eastern parts of Ethiopia. The road is the busiest one in the country, and RTCs are very prevalent. Minibuses, buses, and trucks are abundant on the highway. Collisions in the Addis Ababa and Oromia Regions account for 58% of all fatal collisions and two-thirds of all the injuries in Ethiopia [6]. A retrospective study was conducted on relevant police reports obtained from eight police stations found between Akaki and Adama. The study included reports from July 2007 to June 2012. Both quantitative and qualitative techniques were employed.

### Study population and variables

All RTCs from July 2007 to June 2012 reported in the registry of police stations between Akaki and Adama towns were included in the study, and purposively selected traffic police officers and drivers were used for key informant interview. The dependent variables were the magnitude of RTCs, the trends in RTCs, and the severity of RTCs. The independent variables were age, sex, educational status, driving experience, type of vehicle, type of injury, driver-car relationship (vehicle ownership), level of driver’s license, cause of RTC, light conditions, and weather conditions at the time of the collision.

### Sample size and sampling techniques

All registered RTCs from July 2007 to June 2012 that were documented in police station registries between Akaki and Adama towns were included in the sample. Besides, purposively selected traffic police officers as well as drivers who were used for key informant interview were also included in the sample.

### Data collection process and data quality control

Data were collected from the eight police stations’ RTC registry, using a checklist that was prepared based on the RTC registry format. Eight key informant interviews (four with drivers and four with traffic police officers), based on the RATS guidelines [7], were conducted in Gelan and Dukem towns. Informant drivers and police officers were purposively selected. A content analysis approach was applied to the information obtained from interviewees. The checklist for data collection was first prepared in English and then translated into Afan Oromo and back into English to ensure its consistency. The data collectors and the data collection supervisors were educated about the project’s objectives and trained in the data collection procedures. Every day, after data collection, data were checked for completeness and coherence. Data were cleaned and edited by removing missing values, using the Epi-info Version 3.5.1 statistical package.

### Data processing and analysis

Data were entered and cleaned using Epi-info Version 3.5.1, and then analyzed in SPSS Version 16. Descriptive statistics were used to explore driver-related characteristics and the occurrence of RTCs. Binary logistic regression was used to assess associations between RTCs and driver-related characteristics, collision cause, time, location, and type of vehicle. All variables with *p-value* ≤0.2 in bivariate analysis were retained for multivariable analyses. Adjusted odds ratios (AOR) and 95% confidence intervals (CI) with *P* <0.05 were considered statistically significant. Chi-square tests were used to explore RTCs for linear trends.

The qualitative data were analyzed using a content analysis technique. The transcribed text was imported into OpenCode to facilitate the coding process. Units of relevant meaning were examined line-by-line and coded. As part of the analysis, four sub-themes that show the meaning of our findings were developed. A single theme represents our overall interpretation of the qualitative data.

### Operational definitions

#### Fatal accident

At least one person (driver, passenger or pedestrian) died, within 30 days, from injuries received as a result of an RTC.

#### Severe injury

At least one person was injured and admitted in hospital, but no deaths occured.

#### Slight injury

At least one person required medical care, but no fatalities or injuries that required hospitalization occured.

#### Property damage

All collisions that did not result in injuries or deaths.

#### Driver’s licensing system in Ethiopia

The criteria of licensure for motorcycle or automobile drivers; for tankers, bus or taxi drivers; and truck or special mobile equipment drivers are: should have at least complete fourth grade education and be not less than 18 years of age; should have at least complete an eighth grade education and be not less than 24 years of age; and should have at least complete an eighth grade education and be not less than 20 years of age, respectively. Any person to be eligible for certification of license shall take an integrated theoretical and practical driving training and pass the examination.

#### Isuzu

A vehicle that is used for loading and transporting people (also known as Kitkit in Ethiopia), with a carrying capacity of 27 individuals. It is restructured for the purpose of human transportation in Ethiopia.

### Ethical considerations

The study protocols were approved by the Institutional Ethical Review Committee of Addis Ababa University College of Health Sciences. An official letter of cooperation was written by the School of Public Health (Addis Ababa University) to the Oromia Police Commission and the commission wrote a letter of support to all the police stations in the study area. Information about the study was given to the police officials working on RTCs. Verbal consent was obtained from the police officials, the traffic police officers, and the drivers.

## Results

### Driver-related characteristics

Between July 2007 and June 2012, there were 2,335 RTCs registered in the eight police stations (between Akaki and Adama towns). The outcomes of 24 collisions were not registered. The mean age of the drivers was 32.9 (SD ± 9. 8) and almost all of them were male (99.4%). Only 34.4% and 27.8% of the drivers had completed elementary and high school, respectively. More than half of the collisions (51.7%) were caused by 19–30 year old drivers, and another 40.3% of collisions were caused by 31–50 year old drivers. Some of the drivers (46.7%) had a level three driver’s license and few (20.2%) had level four. About one-third of drivers involved in RTCs had 3–5 years of driving experience, followed by 27.2% of drivers with less than three years of driving experience. RTCs by hired drivers were more common (83.6%) than those by the owners of the vehicle (15%). Among all crashes, 39 (1.7%) occurred in the rain and 346 (15.2%) at night (Table 1).

**Table 1:** **Frequency distribution of driver’s related characteristics involved in RTCs between Akaki and Adama, central Ethiopia from July 2007 to June 2012**

	Frequency	Percent
Age of driver (2274)		
≤18	25	1.1
19-30	1175	51.7
31-50	916	40.3
50+	158	6.9
Sex of driver (2318)		
Female	15	0.6
Male	2303	99.4
Vehicle ownership (2299)		
Owner	344	15
Hired	1923	83.6
Others*	32	1.4
Educational status (2063)		
0-4	74	3.6
5-8	712	34.4
9-10	577	27.8
11-12	497	24
12+	212	10.2
Driving experience (1473)		
<1 year	129	8.7
1-3years	401	27.2
3-5 years	479	32.6
5-10years	345	23.4
10 + years	119	8.1
Driver’s license (2162)		
No driving license	26	1.2
1st level	39	1.8
2nd level	205	9.5
3rd level	1009	46.7
4th level	436	20.2
5th level	432	20
Special license	15	0.7
Vehicle has deficiency (2190)		
Yes	2171	99.1
No	19	0.9
Weather condition (2318)		
Rainy	39	1.7
Dry	2261	97.5
Others**	18	0.8
Light condition (2294)		
Dark midnight	142	6.2
Dark early night/morning	206	9
Daylight	1946	84.8

*Friend, relatives of the owners, ranted **Cloudy, windy.

### Magnitude of RTCs

**Figure 1:**
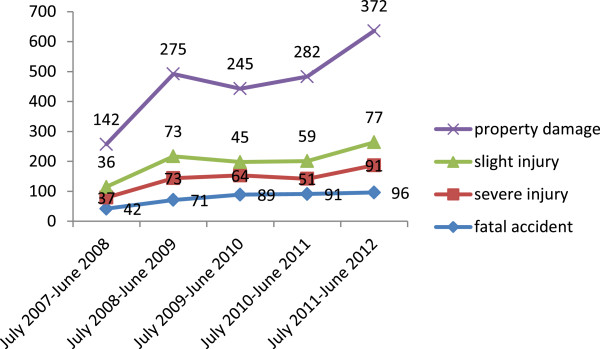
**Trends of Road Traffic Collision from Akaki to Adama, central Ethiopia July 2007 to June 2012.**

A total of 515 people died in 389 (16.7%) of the collisions, while 549 (31.5%) and 681 (39%) people were severely and slightly injured, respectively. In addition, 316 (13.5%) and 290 (12.4%) of the crashes caused severe and slight injuries, respectively. The remaining collisions brought about only property damage. Among those killed in RTCs, 307 (59.9%) were pedestrians, 145 (28.2%) were passengers, and 63 (12.2%) were drivers. Overall, 1,745 people (183 drivers, 750 passengers, and 812 pedestrians) were affected by the crashes. (Figure 1) Over three-quarters of the victims, 1,333 (76.4%), were male.

**Figure 2:**
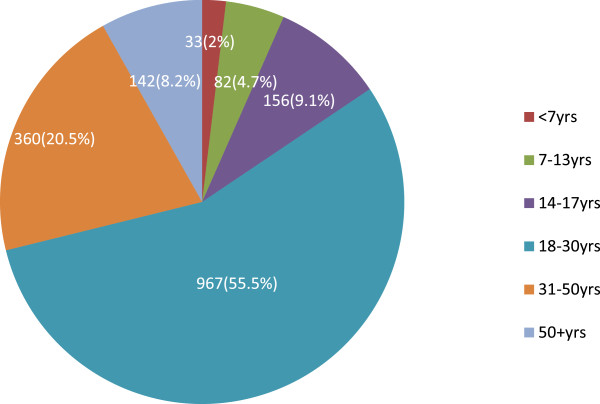
**Age distribution RTCs victims between Akaki and Adama, central Ethiopia from July 2007 to June 2012.**

The number of victims of RTCs increased from 261 to 494 in the five-year period (Table 2). As depicted in Figure 2, 967 (55.5%) victims were in the age group of 18–30 years, followed by 360 (20.5%) in the age group of 31–50 years, and the remainder, 33 (2%), were less than 7 years. 

**Figure 3:**
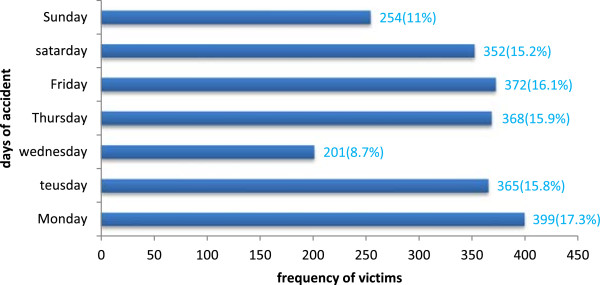
**Distribution of RTCs by day between Akaki and Adama, central Ethiopia from July 2007 to June 2012.**

As presented in Figure 3, 399 (17.3%) collisions occurred on a Monday, and 368 (15.9%) collisions on a Thursday. The fewest collisions, 201 (8.7%), occurred on a Wednesday.

**Table 2:** **Frequency distributions of RTCs victims between Akaki and Adama, central Ethiopia from July 2007 to June 2012**

Year	Frequency of victims’	Percent
July 2007 to June 2008	261	15
July 2008 to June 2009	312	17.8
July 2009 to June 2010	282	16.2
July 2010 to June 2011	396	22.7
July 2011 to June 2012	494	28.3

### Trends of RTCs

**Figure 4:**
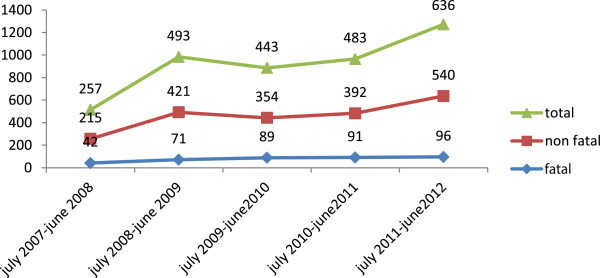
**Trends of fatal and nonfatal RTCs between Akaki and Adama, central Ethiopia from July 2007 to June 2012.**

**Figure 5:**
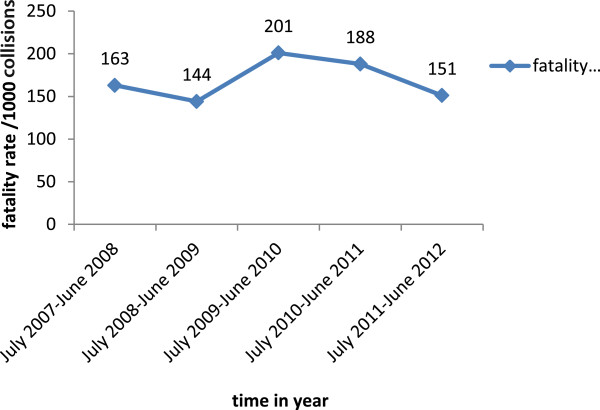
**Fatality rate per 1000 collisions between Akaki and Adama, central Ethiopia from July 2007 to June 2012.**

Two hundred fifty seven (11.1%) RTCs occurred during the period of July 2007 to June 2008. This nearly doubled to 492 (21.3%) during the period of July 2008 to June 2009. However, it showed a slight decrease to 443 (19.2%) between July 2009 and June 2010, compared to July 2008 through June 2009. Between July 2010 and June 2011, there were 483 (20.9%) RTCs, compared to July 2011 through June 2012 during which there were 636 (27.5%) RTCs, showing the highest increment compared to all the other years. Fatal RTCs doubled from 42 (10.8%) to 96 (24.7%) and non-fatal RTCs increased as well from 215 (11.2%) to 540 (28%). The magnitude of RTCs showed an increasing trend during the study period. (Figure 4)The fatality rate per 1000 collisions was the least during the July 2008 to June 2009 period, while it was highest between July 2009 and June 2010. Between July 2010 and June 2012, it showed some tendency of decrease (Figure 5).

#### Factors associated with RTCs

The drivers’ age, sex, education, driving experience, level of driver’s license, and vehicle ownership were not significantly associated with the fatal RTCs in a bivariate analysis. (Table 3) However, location, time of collision (day and night), driving above speed limit, careless driving, type of vehicle, and failing to give priority for other vehicles and pedestrians had a statistically significant association with the fatal RTCs in the bivariate analysis. Some of the nonfatal (44%) and fatal (32.9%) RTCs occurred in Adama town. Twelve percent (12%) and 5% of the fatal and the nonfatal RTCs occurred at night, respectively. Careless driving caused 13.1% and 27.1% of the fatal and the nonfatal RTCs, respectively (Tables 4 and 5).

**Table 3:** **Driver’s related factors associated with RTCs between Akaki and Adama, central Ethiopia from July 2007 to June 2012**

Variables	Outcomes		
	Fatal number (%)	Nonfatal number (%)	p-value
Age of driver (2263)			
Less or equal to18	5(1.4)	20(1.1)	0.592
19-30	193(52.1)	974(51.5)	
31-50	152(41.1)	761(40.2)	
50+	20(5.4)	138(7.3)	
Sex of driver (2307)			
Female	5(1.3)	10(0.5)	0.089
Male	379(98.7)	1913 (99.5)	
Vehicle owner ship (2290)			
Owner	49(12.9)	293 (15.3)	0.366
Hired	323 (85.2)	1593(83.4)	
Others*	7(1.8)	25(1.3)	
Educational status (2063)			
0-4	13(3.8)	61(3.5)	0.326
5-8	127(37.1)	583(33.9)	
9-10	102(29.8)	470 (27.3)	
11-12	71(20.8)	425(24.7)	
12+	29(8.5)	182 (10.6)	
Driving experience (1473)			
<1 yr	21(8)	108(8.9)	0.873
1-2year	79(30)	322(26.6)	
3-5 year	77(29.3)	402(33.2)	
5-10 year	66(25.1)	279(23.1)	
10+ year	20(7.6)	99(8.2)	
Grade of driver’s license (2153)			
No driving license	4(1.1)	22(1.2)	0.843
1st level	6(1.7)	33 (1.8)	
2nd level	30(8.4)	175 (9.7)	
3rd level	174(48.9)	830(46.2)	
4th level	77(21.6)	357(19.9)	
5th level	63(17.7)	368(20.5)	
Special license	2(0.6)	12(0.7)	

*Friend, relatives of the owners, ranted.

**Table 4:** **Characteristics of RTCs according to time, place and Weather condition between Akaki and Adama, central Ethiopia from July 2007 to June 2012**

Variables	Outcomes		
	Fatal number (%)	Nonfatal number (%)	p-value
**Place of collision (2310)**			
Adama	128 (32.9)	847 (44)	0.001
Mojo	87 (22.3)	307 (15.9)	
Bishoftu	84 (21.6)	375 (19.8)	
Dukem	64 (16.5)	269 (13.9)	
Gelan	26 (6.7)	123 (6.4)	
**Year at collision (2335)**			
July 2007 to June 2008	42 (10.8)	215 (11.2)	0.085
July 2008 to June 2009	71 (18.3)	421 (21.9)	
July 2009 to June 2010	89 (22.9)	354 (18.4)	
July 2010 to June 2011	91(23.4)	392 (20.5)	
July 2011 to June 2012	96(24.7)	540 (28)	
**Vehicle has deficiency (2183)**			
Yes	3 (0.8)	15 (0.8)	0.646
No	362 (99.2)	1803 (99.2)	
**Weather condition at the time of the collision (2312)**			
Rainy	5 (1.3)	34 (1.8)	0.804
Dry	379 (97.9)	1876 (97.5)	
Others*	3 (0.8)	15 (0.8)	
**Light condition at the time of collision (2286)**			
Dark midnight	46 (12)	96 (5)	<0.001
Dark early night/morning	50 (13.1)	156 (8.2)	
Daylight	287 (74.9)	1651 (86.8)	

*cloudy, windy.

**Table 5:** **Causes of RTCs and types of car involved in road traffic collision between Akaki and Adama, central Ethiopia from July 2007 to June2012**

Variables	Fatal	Nonfatal	
	Frequency (%)	Frequency (%)	p-value
**Causes of collision (2311)**			
Careless driving	51 (13.1)	522 (27.1)	< 0.001
Over speeding	185 (47.8)	651 (33.9)	
Failure to give priority	101 (26)	406 (21.1)	
Vehicle defect	10 (2.6)	10 (0.5)	
Road problem	1 (0.3)	4 (0.2)	
Pedestrian error	1 (0.3)	6 (0.3)	
Follow too closely	13 (3.3)	249 (12.9)	
Unidentified causes	22 (5.7)	45 (2.3)	
Others*	5 (1.3)	29 (1.5)	
**Type of vehicle (2185)**			
Cycle/motor cycle/ Bajaj	24 (6.6)	146 (8)	0.201
Minibus	55 (15)	264 (14.5)	
Bus	19 (5.2)	87 (4.8)	
Isuzu (people)	19 (5.2)	72 (4)	
Automobiles	36 (9.8)	181 (10)	
Trucks	89 (24.3)	516 (28.4)	
Isuzu (loading)	85 (23.2)	315 (17.3)	
Pick up and Toyota	27 (7.4)	172 (9.5)	
Land cruiser	7 (1.9)	48 (2.6)	
Others**	5 (1.3)	18 (1)	

*cattle’s, horses running and failing under vehicles **like: coaster, cart,

In a logistic regression model, it was found that RTCs caused by driving above the speed limit (AOR = 5.3, 95% CI: 2.9, 9.6) and failing to give priority for other vehicles and pedestrians (AOR = 5, 95% CI: 2.3, 9.3) were significantly associated with RTC fatality. Furthermore, fatal collisions were more likely to occur in vehicles that had a defect (AOR = 19, 95% CI: 6.4, 56) and with unidentified causes of the collision (AOR = 8.3, 95% CI: 3.6, 18.8). Careless driving (AOR = 1.78, 95% CI: 0.94, 3.4) and pedestrian errors (AOR = 3.9, 95% CI: 0.4, 35) also increased fatal collisions although not statistically significant. The nighttime RTCs were 2.5 times (AOR = 2.5, 95% CI: 1.7, 3.7) more likely to be fatal than RTCs that occurred at daytime (Table 6).

**Table 6:** **Association of fatal RTCs with selected risk factors; multivariable-adjusted odds ratios (OR) from the binary logistic regression**

Variables	Outcome		Crude OR	Adjusted OR
	Fatal number (%)	Nonfatal number (%)		
**Place**				
Adama	128 (32.9)	849 (44)	1 (reference)	1 (reference)
Mojo	87 (22.4)	307 (15.9)	1.9 [1.4-2.5]**	1.36[0.97, 1.9]
Bishoftu	84 (21.6)	301 (19.8)	1.5 [1.03-1.9]*	1.26[0.9, 1.77]
Dukem	64 (16.5)	269 (13.9)	1.6[1.1-2.2]*	1.64[1.1, 2.3]*
Gelan	26 (6.7)	123 (6.4)	1.4 [0.9-2.2]	1.6 [0.97-2.6]
**Year**				
July 2007 to June 2008	42 (10.8)	215 (11.2)	1 (reference)	1 (reference)
July 2008 to June 2009	71 (18.3)	421 (21.9)	0.8[0.6-1.3]	1.08[0.7-1.7]
July 2008 to June 2010	89 (22.9)	354 (18.4)	1.3[0.9-1.9]	1.75[1.1-2.7]*
July 2010 to June 2011	91 (23.4)	393 (20.5)	1.2[0.8-1.8]	1.5[0.98-2.3]
July 2011 to June 2012	96 (24.7)	540 (28)	0.91[0.6-1.4]	1.27[0.9-1.9]
**Light condition**				
Dark (midnight)	46 (12)	96 (5)	2.8[1.9-4]**	2.5[1.7-3.7]**
Dark (early night/morning)	50 (13.1)	158 (8.2)	1.8[1.3-2.6]**	1.67[1.2-2.4]*
Day light	278 (74.9)	1651 (86.8)	1 (reference)	1 (reference)
**Sex**				
Female	5 (1.3)	10 (0.5)	2.5[0.9-7.4]	4.74[1.5-15]*
Male	379 (98.7)	1913 (99.5)	(reference)	1 (reference)
**Cause of collision**				
Careless driving	51 (13.1)	522 (27.1)	1.87[0.99-3.5]	1.78[0.9-3.4]
Driving above speed limit	185 (47.6)	651 (33.8)	5.4[3–9.7]**	5.3[2.9-9.6]**
Failing to give priority	101 (26)	406 (21.1)	4.76[2.6-8.6]**	5 [2.3-9.3] **
Vehicle defect	10 (2.6)	10 (0.5)	19.2[6.8-54]**	19[6.4-56]**
Road defect	1 (0.3)	4 (0.2)	4.78[0.5-46]	2.9[0.3-29]
Pedestrian error	1 (0.3)	6 (0.3)	3.19[0.36-28.5]	3.9[0.4-35]
Unknown cause	22 (5.7)	45 (2.3)	′9.36[4.4-20]**	8.3[3.6-18.8]**
Others***	5 (1.3)	33 (1.7)	2.9[0.97-8.7]	2.3[0.8-7.2]
Following too closely	13 (3.3)	249 (12.9)	1 (reference)	1 (reference)

Overall *P* value *p <0.05 **p ≤0.001 ***cattle’s, horses running and failing under vehicles.

### Results from Qualitative finding

The results presented below summarize the responses of drivers and traffic police officers. Respondents’ views about the overall situation of RTCs in the study area, causes and contributing factors for RTCs and its trend, possible reasons for drivers to exercise risky driving behaviors, pedestrians and road related factors for RTCs were assessed. Participants felt that economic problems, pedestrian ignorance and the current mismatch of vehicle fleet and existing road conditions are the major contributors of RTCs and increasing trend (Table 7).

**Table 7:** **Theme: Road traffic collision is caused by a road user problem and aggravated by different factors**

Categories	Young age drivers are most frequently involved in RTCs	Trend of RTC is increased due to the incompatibility of vehicle flow and condition of the road	RTCs are mainly caused by drivers’ errors and aggravated by economic problems and other factors	Pedestrian ignorance is huge problem leading to the accident
**Codes**	High speed	Carelessness	Economical problem	Carelessness
	Improper turning	High speed	High vehicle fleet	Low awareness
	Substance abuse	Road problem	Miss match	Ignorance
	Sleep disturbance	Improper inspection	Licensing issues	Irresponsible
	Working illegally	Sleep disturbance	Light condition	Doing forbidden
	Fail to give priority	Irresponsible	Weather condition	
	Carelessness	Fail to give priority	Substance abuse	
	Greediness	Releasing strong light	Driving at night	
		High vehicle fleet		
		Miss match		

### Theme: Road traffic collisions are caused by a road user problem and aggravated by different factors

#### Subtheme 1. Young age drivers are most frequently involved in RTCs

According to the qualitative informants, young drivers are driving irresponsibly and carelessly because they have nothing to worry about and are less experienced. Most young drivers have a third level driver’s license and are frequently involved in the collision. Older drivers have family responsibilities and most of them drive their own vehicles. Therefore, older drivers drive more carefully which decreases their risk of collision.

Participant 2, a traffic policeman insisted, “*currently drivers who cause RTCs are young ones. Now, if you see drivers of minibus and Isuzu, they are very young and even you doubt that they have driver’s license. Those who have third level driver’s license are frequently involved in crashes. They don’t care; they don’t feel responsible. They drive too fast”*

#### Subtheme 2. Trend of RTC is increased due to the incompatibility of vehicle flow and condition of the road

The trend of RTC was increasing in the study area because the number of vehicle fleet was increasing from time to time and the increment was beyond the capacity of the road.

Participant 1, a traffic policeman responded that “*…the number of vehicle flow increased every year and not compatible with existing road condition.”*

#### Subtheme 3. RTCs are mainly caused by drivers’ errors and aggravated by economic problems and other factors

Most of the RTCs were caused by drivers’ errors. The main errors were driving too fast, driving dangerously close to another vehicle, turning improperly, and failure to give priority for pedestrians and other vehicles.

Participant 1, a traffic policeman commented, “*the drivers are responsible for the occurrence of the collision because they can prevent collisions by adjusting themselves to the existing situation. If the road is inconvenient, they should drive slowly and carefully. They should keep the required distance while following another car.”*

Most of the drivers were hired and they tried to do more than what was expected from them to get extra money. In many cases, they were driving for more than 24 hours. In order to avoid sleep, they used different substances like khat and shisha, which made them over-stimulated and led them to reckless driving which was the main cause of the collision.

Participant 6, a driver claimed, “These days’ *minibus and Isuzu drivers are frequently causing collisions. They simply think about the money. They don’t care about anything else. They are moving at night. So if drivers are driving without sleeping, they use khat and shisha to avoid sleep. The reason for over speeding is about the benefit. If you ordered him to do something, he rushes to have extra money for himself. He rushes to drop the first and to be the first in the next turn.”*

#### Subtheme 4. Pedestrians’ ignorance is a big problem

Many pedestrians in the towns are not abiding by the traffic rules and they are not moving in the appropriate direction. They are ignorant about road safety rules. But some pedestrians have a little knowledge of the traffic rules and they often walk on the right side of a road.

Participant 8, one driver stated “*in the towns, pedestrians do not feel responsible. They fear an ox more than a vehicle. Some stand and greet each other in the middle of a road. Some even behave as if they had a right to sleep on the zebra road. Really they are ignorant.”*

## Discussion

RTCs have become a major public health and economic problem worldwide. This problem is getting worse in developing countries, like Ethiopia. Our study showed that many (n = 2335) RTCs occurred on the road between Akaki and Adama towns from July 2007 to June 2012 and the incidence is increasing. Location of the collision, light conditions at the time of collision, driving above the speed limit, failing to give priority for other vehicles and pedestrians and vehicular technical problems are strong determinants of road traffic fatality.

The RTC fatality rate (16.7%) found in this study is lower than the national fatality rate in Ethiopia (22%) [8]. This difference might be due to the small sample of the country our study covered. Thus, it could underestimate the result. From July 2007 to June 2008, 257 (11.1%) collisions occurred. Within five years, this collision rate nearly tripled for the period of July 2011 through June 2012 (637, 27.5% collisions). Similar to this study, a Dubai cross-sectional study between 2002 and 2008 [9] and a Lithuania time trends study from 1998 to 2007 [10] reported a steadily increasing number of RTCs. The trend of RTCs in our study revealed an increasing pattern, but it was not statistically significant. The main reasons for the increase could be the increase in number of vehicles on the route and the small size of the road. Moreover, poor vehicle technical inspections and poor enforcement of traffic safety rules in the country could aggravate the problem. High numbers of RTCs were registered at Adama, 977 (42%). Adama is highly populated and there are a large number of vehicle fleets in the town, possibly accounting for the high frequency of RTCs. However, fatal collisions were 64% more likely in Dukem than in Adama. Unlike other similar studies which reported more RTCs made by 31–40 years of age drivers [11, 12], and more fatal accidents by older drivers [13], in our study, more than half of the collisions (51.6%) were caused by young drivers (19–30 years of age), and age did not have statistically significant association with fatal RTCs. This is consistent with findings from other similar studies [9, 13–16]. The lack of experience of young drivers, as well as their high risk-taking behavior could be the reason for their dominance. In this study, like similar investigations done elsewhere [16–18], almost all of the drivers that caused crashes were male (99.6%). This preponderance of males in collisions might be due to greater involvement of males, compared to females, in professional driving activities. A study in Qatar reported more RTCs (66.7%) by drivers who owned the vehicles than by those who were hired [16], but 83.7% of the crashes examined in this study were made by hired drivers. This discrepancy could arise from the high socioeconomic difference between the population of Ethiopia and of Qatar.

More than 93% of the collisions were caused by driver errors, such as driving carelessly, failing to give priority to other vehicles and pedestrians, driving above the speed limit and following too closely. A study in Kenya also showed that 85% of the collisions were caused by human factors (driver and pedestrian errors) [16]. High speeds and failure to give priority caused five times more fatal collisions than following too closely, supplementing the existing literatures [1, 12, 19–21]. Furthermore, most fatal road collisions were caused by vehicles with some defect, unidentified causes of collisions, and careless driving.

Alcohol was never reported as a cause of RTCs in our study. However, many studies claimed alcohol as a major cause of crashes and fatalities [10, 15, 22, 23]. Lack of information about drivers’ alcohol consumption in the registry of the police stations could explain the variability.

In our study, similar to one in Tanzania [17], more of the fatal RTCs happened at night, rather than during the day. This is probably because, at night, drivers are operating vehicles under the influence of shisha, khat, and/or fatigue, which negatively affects their concentration and alertness [24]. Moreover, the traffic policemen do not work after 8:00 pm. This reduces supervision and delays rescue and provision of first aid to victims. Trucks, Isuzus (loading) and minibuses were most frequently involved in the crashes. This is inconsistent with studies in Kenya [25] and Qatar [16] where cars, pick-up trucks and vans were most frequently involved in crashes. In this study, driving experience was not a determinant factor for fatal collisions. However, drivers with only 3–5 years’ experience were frequently involved in the crashes. A study in Qatar noted that drivers who drove for more than five years were more frequently involved in the crashes [16]. Although driving experience was not significantly associated in this study, literature revealed that fatality was highly associated with risky driving behaviors [17, 26]. There were controversial findings that having more driving experience was associated with risky behaviors, which was the determinant of fatality [26]. In contrast, a study in Tanzania showed that less experienced drivers were found to have high risky driving behaviors [17]. Unlike the above studies, driving experience was not found to be a predictor variable for risky driving behaviors [18].

The level of driver’s license and educational status of the drivers were not predictors of fatality in this study. Drivers who completed primary levels of education (5–8 grade) caused a large number, 712(34.4%), of RTCs and a study in Addis Ababa revealed similar results [14]. In a similar fashion, having secondary educational levels or above increased risk of RTCs in some studies [14, 17]. 

This is supported also by a study in Qatar in which the highest frequency of crashes (32.7%) occurred among drivers who had university degree [16]. A valid driving license and years of driving experiences were protective for the occurrence of the collisions [27].

Out of total victims, 550(29.5%) were fatally injured, 549(31.5%) were severely injured and 681(39%) were slightly injured. This was quite different from a study in Kenya where only 10% resulted in fatalities [25]. In our study, 88% of the fatalities were pedestrians and passengers. This is a similar finding to a study conducted in the geographic regions between Addis Ababa and Shashamane in which fatalities were 91% [28]. In addition, a Kenyan and Cameroon studies showed nearly similar percentages of the fatalities, 80% and 74%, respectively [29]. However, urban environments in Ethiopia do not have proper pedestrian facilities. Additionally, the road users are not fully informed of traffic rules and regulations in the country. This could explain the slight difference. In contrast, a study in Brazil revealed that drivers accounted for 67.8% of deaths [30]. This was supported by studies in which frequent victims of RTCs in highly-motorized countries and low-income countries, respectively, are car drivers and occupants of multiple passenger vehicles (such as buses) and pedestrians [31, 32]. In our study, the majority of the victims were males, aged 18–30 years, which is similar with another study [33]. The dominance of males and younger age groups might be due to their frequent movements from town to town for different businesses. Moreover, younger men are more likely to engage in risky behaviors like running red lights, violating traffic signs and signals, and passing dangerously.

Occupational road safety is a shared responsibility between owners of vehicles and drivers. Owners of the cars have the responsibility for the performance of their drivers and control of every driving activity of drivers while they are on the road. The individual drivers also have a legal obligation to behave safely, and not to endanger themselves or others. But in Ethiopia there is no road safety policy despite the high toll of road traffic casualties in the country.

This study has some limitations. Lack of some very useful data was a problem. For example, the records in the police stations said nothing about the utilization of seatbelt and helmets, as well as the drivers’ alcohol use. We were also unable to get valid information about the population at risk, number of vehicles and vehicle fleet of the study area.

## Conclusion

A large number of road traffic collisions occurred, resulting in high mortality and morbidity in the study areas. The trend of road traffic collisions between Akaki and Adama from July 2007 to June 2012 showed increasing patterns for both fatal and nonfatal collisions. Nevertheless, the increment was not statistically significant. Younger ages, males and hired drivers were largely involved in road traffic crashes. This study demonstrated a strong positive association between fatal road traffic collisions and location of collision, time of collision (day or night), high speeds, failure to give priority and vehicular technical problems.

### Recommendations

1. More efforts should be made to enforce young drivers obey traffic rules and enforcement of the speed limit.
2. Driving public transports (vehicles carrying passengers) at night need to be banned and important controlling mechanisms should be arranged.
3. Awareness campaigns on road safety rules should target pedestrians and strengthen the measures taken on pedestrians who don’t abide road safety rules.
4. The country should have a road traffic safety policy.
5. Further large prospective studies are needed to identify the possible factors associated with RTCs.

## Authors’ information

Demeke Assefa and Gezahegn Tesfaye Co-author.

## Abbreviations

AOR: Adjusted odds ratio
CI: Confidence interval
COR: Crude odds ratio
RTC: Road Traffic collision
SPSS: Statistical Package for Social Science
WHO: World Health Organizations.
